# Sigmoid Sinus Wall Reconstruction for Pulsatile Tinnitus Caused by Sigmoid Sinus Wall Dehiscence: A Single-Center Experience

**DOI:** 10.1371/journal.pone.0164728

**Published:** 2016-10-13

**Authors:** Rong Zeng, Guo-Peng Wang, Zhao-Hui Liu, Xi-Hong Liang, Peng-Fei Zhao, Zhen-Chang Wang, Shu-Sheng Gong

**Affiliations:** 1 Department of Otolaryngology Head and Neck Surgery, Beijing Friendship Hospital, Capital Medical University, Beijing, China; 2 Department of Otolaryngology Head and Neck Surgery, Beijing Tongren Hospital, Capital Medical University, Beijing, China; 3 Department of Radiology, Beijing Tongren Hospital, Capital Medical University, Beijing, China; 4 Department of Radiology, Beijing Friendship Hospital, Capital Medical University, Beijing, China; Universita degli Studi di Napoli Federico II, ITALY

## Abstract

**Objective:**

To evaluate clinical characteristics and present surgical outcomes of PT caused by sigmoid sinus wall dehiscence (SSWD)

**Methods:**

This study retrospectively reviewed 34 patients with PT who were diagnosed with SSWD in our institution between December 2008 and July 2013. Among them, 27 patients underwent sigmoid sinus wall reconstruction (surgery group) and 7 patients refused surgery (non-surgery group). Preoperative data were obtained from the patients’ medical records. All patients were followed up regularly for at least 25 months. Preoperative and postoperative computed tomography angiography (CTA) images were compared. Student’s *t*-tests were used to compare age, body mass index (BMI) and preoperative Tinnitus Handicap Inventory (THI) scores between the surgery and the non-surgery groups and to compare pre- and follow-up THI scores.

**Results:**

There was no significant difference in age, body mass index, or preoperative THI scores between groups. Following surgery, 14 patients had complete resolution, 5 had partial resolution, 7 experienced no change and PT was aggravated in 1 patient. The difference between preoperative and postoperative THI scores was significant. No severe complications were found postoperatively. Comparison of the preoperative and postoperative CTA images revealed that remnant unrepaired dehiscences were the cause of unsatisfactory outcomes following surgery. In the non-surgery group, PT remained largely unchanged.

**Conclusions:**

Sigmoid sinus wall reconstruction is an effective and safe treatment for PT caused by SSWD. It is imperative that all regions of the dehiscence are sufficiently exposed and resurfaced during surgery.

## Introduction

Pulsatile tinnitus (PT) is characterised by a rhythmic pulsing often in time with the heartbeat and is caused primarily by vascular anomalies [1, 2]. PT constitutes approximately 4% of all tinnitus [2, 3], and many of them report a significant impact on daily life. Sigmoid sinus anomalies, which include sigmoid sinus diverticulum (SSD) and sigmoid sinus wall dehiscence (SSWD), have recently been found to be common causes of PT [4–6]. The prevalence of sigmoid sinus anomalies in PT patients is about 20% [4–6]. These sinus abnormalities have received increasing attention because PT caused by SSD and SSWD is curable with a high rate of success [7–9].

SSD occurs when the sigmoid sinus wall locally intrudes into the mastoid cells or cortex [7, 10], whereas SSWD, also known as sigmoid plate dehiscence [11, 12], refers to a local bone wall defect that leads to direct contact between the sigmoid sinus and surrounding mastoid cells [6, 7, 13]. SSD and SSWD are closely related and often occur concurrently in patients with PT; however, each may be the sole cause of PT [14]. Although both SSD and SSWD occur in non-PT patients [5, 6], the prevalence of SSD is lower than that of SSWD [5]. The reason for this difference is not known.

The PT pathogenesis caused by SSWD and SSD differs [14]. PT associated with SSD is caused by turbulent blood flow in abnormal vascular structures, whereas that associated with SSWD is thought to result from the vibration of an uncovered sinus wall and the absence of shielding against the sound of the sinus wall. In patients with SSWD and a dominant sigmoid sinus (DSS), the turbulence of the blood flow may contribute to PT [14]. Furthermore, it is not known whether SSD and SSWD are different stages along a single pathophysiological spectrum or if they represent two individual entities [8]. Evaluation of SSD and SSWD cases separately will help clarify their individual contributions to the pathogenesis of PT.

Sigmoid sinus wall reconstruction and endovascular treatment are effective approaches for the management of SSD [7, 13, 15–18]. Endovascular treatment is not effective for PT caused by SSWD because digital subtraction angiography (DSA) shows no vascular anomaly in many patients [14]. In 2011, Eisenman [7] reported that sigmoid sinus wall reconstruction resulted in complete resolution of PT caused by SSWD, and several subsequent studies have confirmed the author’s findings [8, 13, 19–21]. Although these findings suggest that sigmoid sinus wall reconstruction is an effective treatment for PT caused by SSWD, the sample sizes in these studies were small and some of the cases were non-responders [8]. Thus, further study to refine the surgical technique and evaluate the long-term effects and complications of sigmoid sinus wall reconstruction surgery is warranted.

In our previous study, surgical outcomes of PT caused by SSD were reported and surgical skills were summarized [9]. In the present consecutive study, we focused on PT caused by SSWD. We retrospectively evaluated the clinical features in 34 patients with SSWD and presented surgical outcomes. The pre- and postoperative computed tomography angiography (CTA) findings were analyzed and surgery experience was provided.

## Materials and Methods

### Ethic Statement

This retrospective study was conducted at Beijing Tongren Hospital, Capital Medical University of China. Ethical approval was obtained from the institution’s review board of Beijing Tongren Hospital, Capital Medical University of China. And the methods were carried out in accordance with the Declaration of Helsinki. Patient confidentiality was protected. Their information was anonymized and de-identified before analysis.

### Patient selection

Our retrospective study included patients with PT who were diagnosed with SSWD between December 2008 and July 2013 in the Department of Otolaryngology Head and Neck Surgery at Beijing Tongren Hospital. SSWD was identified on CTA and DSA images as a local dehiscence on the sigmoid sinus bony wall in at least two continuous images in both axial and coronal CTA views [14]. The CTA, also known as dual-phase contrast-enhanced CT or CT arteriography/venography, and DSA protocols have been described previously [11, 14, 22]. Patients who had both SSWD and SSD, and those with other definite causes of PT, were excluded from the study. For consistency, only patients whose surgery had been performed by the same surgeon (Dr. Gong SS) were included in the study. We enrolled a total of 34 patients, 27 of whom opted to undergo surgical sigmoid sinus wall reconstruction (surgery group) and 7 who refused surgery (non-surgery group). These patients overlapped with the SSWD patients in our previous study [14]; however, no repeated results were presented in the present study.

### Preoperative data collection

Preoperative data, including age, sex, body mass index (BMI), medical history, physical examination and CTA and DSA findings, were obtained from the patients’ medical records. CTA and DSA images were examined for dehiscence and sigmoid sinus morphology. Cases of DSS, a sudden increase in the diameter of the sigmoid sinus discordant with adjacent venous sinuses [14], were identified using DSA. Previous studies have referred to DSS as a prominent sigmoid sinus, sigmoid sinus enlargement, sigmoid sinus ectasia, or distensible sigmoid sinus [14, 23–26]. The Tinnitus Handicap Inventory (THI), widely endorsed clinically for quantifying the impact of tinnitus on daily life [27–29], was used to assess the severity of tinnitus.

### Surgery procedures

**Fig 1:**
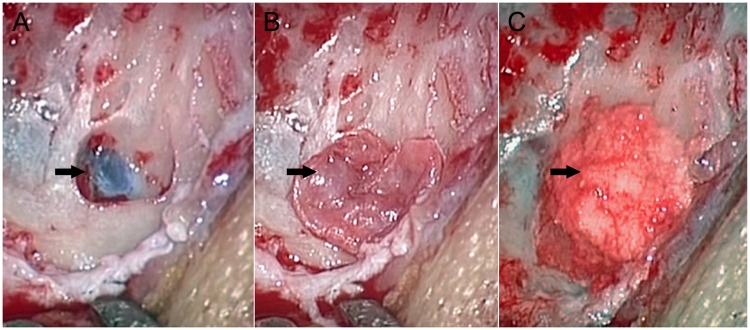
Intraoperative photographs of a patient (Patient 13) with complete resolution of PT following surgery. (A) The exposed dehiscence (arrowhead). (B) The dehiscence covered by the temporalis fascia (arrowhead). (C) The autologous bone powder overlays the temporalis fascia and are fixed using a medical adhesive (arrowhead).

Our surgical sigmoid sinus wall reconstruction protocol was similar to that described in previous studies with amendments aiming at resurfacing the dehiscent bone wall [7, 9, 17, 19]. In brief, under general anaesthesia, a postauricular incision was performed and a pedicle myoperiosteal flap was made to expose the mastoid cortex, and the temporalis fascia was harvested. As shown in Fig 1, the mastoid was opened and, based on CTA images, the dehiscent region of the sigmoid sinus was exposed and skeletonised. Autologous bone powders were collected during the above steps. The temporalis fascia and autologous bone powders were used to resurface the dehiscent area in sequence, and the graft was fixed with a medical adhesive (FAL, Beijing Fuaile Co., Beijing, China). Finally, the mastoid was covered with the pedicle myoperiosteal flap, and the skin incision was sutured. After surgery, a compressive mastoid dressing was applied.

### Surgical outcome evaluation

The patients were followed up regularly for at least 25 months by telephone or clinic visits. Based on the patients’ subjective reports, the surgical outcomes were classified as complete resolution, partial resolution, no change or aggravation of the PT. THI scores were used to assess the patients’ condition at follow-up, and surgical complications were recorded. Only patients who returned to the clinic for follow-up underwent pure tone audiometry and postoperative CTA. Two experienced radiologists who were blind to the patients’ situations analysed pre- and post-operative CTA images separately, and they made the final findings in consensus. They counted the number and measured the diameter of SSWD in reformatted axial images as previously described [11]. In brief, axial images were reconstructed parallel to the horizontal semicircular canal with 1 mm in thickness and increment. It was considered to be SSWD only when the dehiscent wall was found on at least two continuous reformatted axial images. The whole area of the SSWD was the sum of the dehiscent area of each layer, which was approximately equal to the dehiscent diameter of each layer multiplied by the thickness of the layer (1 mm).

### Statistical analysis

All statistical tests were conducted using the SPSS for Windows software (ver. 17.0; SPSS Inc., Chicago, IL, USA). Student’s *t*-tests were used to compare age, BMI and preoperative THI scores between the surgery and the non-surgery groups and to compare pre- and follow-up THI scores. *P*-values < 0.05 were taken to indicate statistical significance.

## Results

### Clinical features

**Fig 2:**
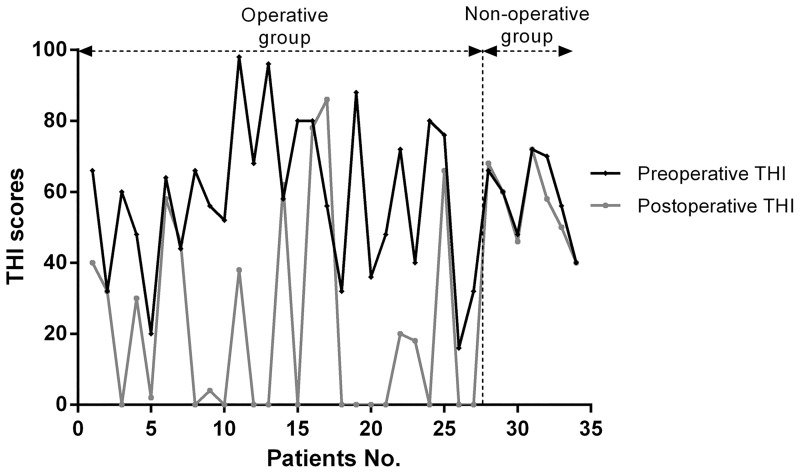
Tinnitus Handicap Inventory (THI) scores. The black and grey spots denote the preoperative and follow-up THI scores, respectively. The vertical dotted line separates the surgery (Patients 1–27) and non-surgery (Patients 28–34) groups.

Patient characteristics are shown in Table 1. The study population consisted of 34 patients of Han Chinese nationality. The surgery group included 27 (25 females and 2 males) patients and the non-surgery group comprised 7 female patients. All patients had unilateral PT that was synchronous with the heartbeat. The average age of participants suffering from PT was 33.24± 10.12 years (range: 18–61 years) and did not differ between groups (surgery, 33.05 ± 10.20 years vs. non-surgery, 34.00 ± 10.58 years; *P* = 0.828). The average BMI was 24.01 ± 2.69 kg/m^2^ (range: 16.85–30.86 kg/m^2^) and was not significantly different between groups (surgery, 24.21 ± 2.94 vs. non-surgery, 23.21 ± 1.16 kg/m^2^; *P* = 0.386). Otoscopic findings were normal for all patients. Compression of the ipsilateral internal jugular vein completely resolved PT in the majority of patients (31/34) and reduced it in the remaining 3 patients. The pure tone audiogram revealed normal hearing at all frequencies in the majority of patients (24/34); however, 10 cases had discrete sensorineural hearing loss at one or more frequencies. SSWD was confirmed by CTA and DSA findings showing bone defects in the sigmoid sinus wall without a diverticulum. DSS was found in 20 patients. The follow-up THI scores did not differ between groups (surgery, 57.93 ± 22.07 vs. non-surgery, 58.86 ± 11.77; *P* = 0.916; Fig 2).

**Table 1:** Patient characteristics.

Patient Number ^a^	Age of onset (years)	BMI(kg/m^2^)	DSS	Manner of follow up	Surgical outcome	Loudness (%)^d^
1	33.5	24.14	N^b^	Telephone	Partial resolution	50
2	54	23.42	N	Clinic	No change	100
3	19	16.85	N	Telephone	Complete resolution	0
4	38	21.79	N	Clinic	Partial resolution	30
5	41.25	24.22	N	Clinic	Complete resolution	0
6	26	25.24	Y^c^	Clinic	No change	100
7	36	22.03	Y	Clinic	No change	90
8	32.5	26.12	Y	Telephone	Complete resolution	0
9	29	26.67	Y	Clinic	Partial resolution	50
10	35	24.22	Y	Telephone	Complete resolution	0
11	36	26.96	N	Clinic	Partial resolution	30
12	19.5	23.62	N	Telephone	Complete resolution	0
13	32	24.77	Y	Clinic	Complete resolution	0
14	31	28.31	N	Telephone	No change	100
15	45	30.86	Y	Clinic	Complete resolution	0
16	37	25.91	N	Clinic	No change	100
17	61	24.84	N	Telephone	Aggravation	150
18	44	25.63	N	Telephone	Complete resolution	0
19	33	23.15	N	Clinic	Complete resolution	0
20	37	28.73	Y	Telephone	Complete resolution	0
21	18	23.88	Y	Telephone	Complete resolution	0
22	29.5	19.92	Y	Clinic	Partial resolution	60
23	19	22.48	Y	Clinic	No change	90
24	27	21.67	Y	Telephone	Complete resolution	0
25	28	19.92	Y	Clinic	No change	100
26	28	25.91	Y	Telephone	Complete resolution	0
27	23	22.48	Y	Telephone	Complete resolution	0
28	26	25.00	N	Telephone	No change	100
29	21.5	22.59	Y	Telephone	No change	100
30	27.5	21.36	Y	Telephone	No change	100
31	30	23.98	Y	Telephone	No change	100
32	50	23.54	N	Telephone	No change	90
33	38	23.33	Y	Telephone	No change	100
34	45	22.66	Y	Telephone	No change	100

BMI, body mass index; DSS, dominant sigmoid sinus in ipsilateral pulsatile tinnitus (PT)

^a^ Patients 1 to 27 and Patients 28 to 34 belong to the surgery and non-surgery groups, respectively

^b^ N represents “no distensible sigmoid sinus”

^c^ Y represents “distensible sigmoid sinus”

^d^ The loudness level in postoperative PT (or follow up PT) compared with that of the preoperative PT (%).

### Intraoperative findings

One or more sigmoid sinus wall dehiscences were found in 25 of the 27 patients who underwent surgery. The dehiscent segment of the sigmoid sinus wall was generally found in the upper lateral portion of the sigmoid sinus (Fig 1). The exposed sigmoid sinus in the dehiscent region beat rhythmically in several patients. In contrast to the other patients, we found a thin bone plate rather than a dehiscence in the sigmoid sinus wall of Patient 17. Additionally, no obvious dehiscence or thin bone plate was detected in Patient 2.

### Surgical outcomes and complications

The average follow-up time was 44.96 ± 12.74 months (range: 25–67 months). In the surgery group, 14 patients returned to the clinic for follow-up visits and underwent a postoperative CTA and hearing test, whereas the remaining 13 patients were followed up by telephone. The pre- and postoperative THI scores were significantly different in the surgery group (preoperative, 57.93 ± 22.07 vs. postoperative, 21.48 ± 28.02; *P*<0.01; Fig 2).

Following surgery, 14 patients achieved complete resolution, 5 had partial resolution, 7 patients reported no change and PT was aggravated in 1 patient. Two patients (Patients 7 and 23) with postoperative PT loudness percentages of 90% were judged to have experienced no change. We found no significant differences in age (*P* = 0.844), BMI (*P =* 0.908) or preoperative THI score (*P* = 0.559) among patients with complete resolution, partial resolution and no change in the condition. The patient whose PT was aggravated was excluded from the analysis due to the limitations of a small sample size.

The association between surgical outcome and the sigmoid sinus morphology is shown in Table 2. A total of 9 patients (9/15, 60%) with DSS and 5 patients (5/12, 41.67%) with a normal sigmoid sinus achieved complete resolution.

**Table 2:** Association between surgical outcome and sigmoid sinus morphology.

	Complete resolution	Partial resolution	No change	Aggravation	Total
**Normal**	5	3	3	1	12
**DSS**	9	2	4	0	15
**Total**	14	5	7	1	27

DSS, dominant sigmoid sinus.

Most of the patients who underwent surgery had no major complications; however, a few patients reported minor complications, including persistent periauricular numbness (Patients 6, 15, 18, 19 and 20), ear fullness (Patients 5, 6, 11 and 20) and collapse of the retroauricular area (Patients 5 and 16). No postoperative hearing loss, headache, dizziness, or visual changes were reported.

All of the patients in the non-surgery group were followed up by telephone. All of them had no change in PT (Table 1). We found no significant difference between the preoperative and follow-up THI scores in the non-surgery group (preoperative, 58.86 ± 11.77 vs. follow-up, 56.29 ± 11.63; *P* = 0.21; Fig 2).

### Pre- and postoperative CTA findings

**Fig 3:**
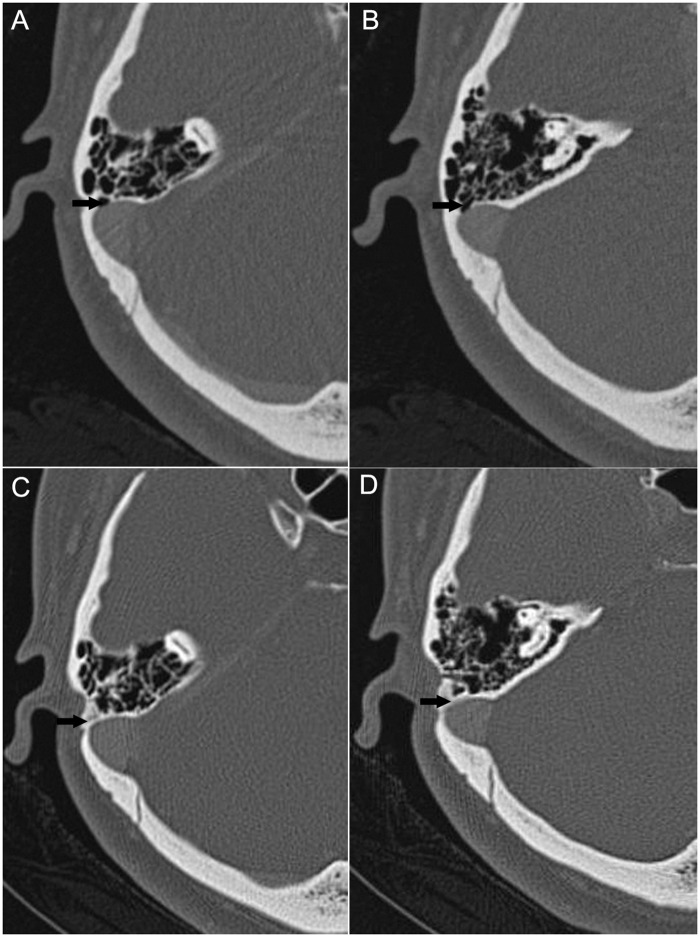
Preoperative (A, B) and postoperative (C, D) CTA findings in a patient (Patient 13) with complete resolution of PT following surgery. The layers in (A) and (B) correspond to those in (C) and (D), respectively. The dehiscences are completely repaired in the two layers postoperatively (arrowheads).

Four patients who had complete resolution of PT following surgery returned to clinic for follow-up. A comparison of the pre- and postoperative CTA images revealed that the dehiscence was completely resurfaced in these cases (Fig 3).

**Fig 4:**
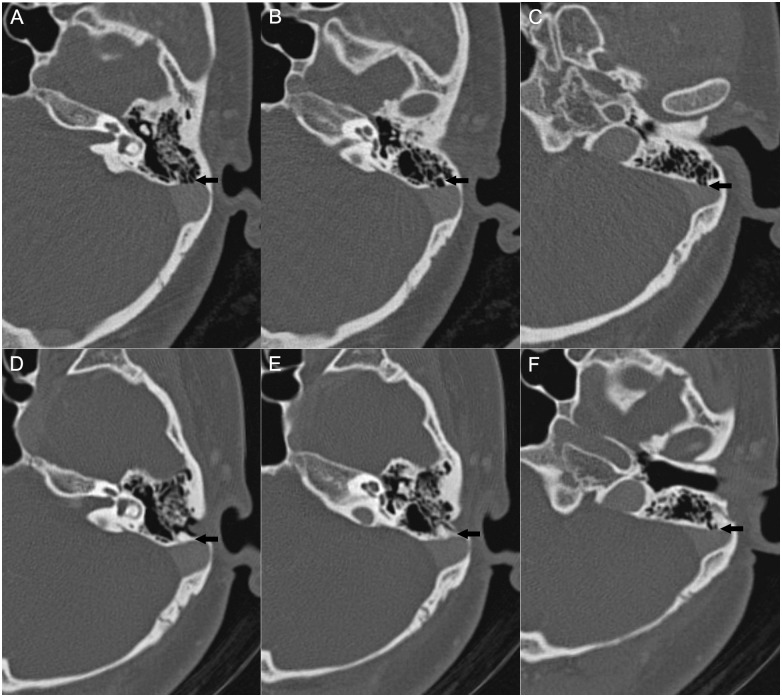
Preoperative (A–C) and postoperative (D–F) CTA images of a patient (Patient 9) with partial resolution of PT following surgery. The layers in (A), (B) and (C) correspond to those in (D), (E) and (F), respectively. The dehiscences in layers (A) and (B) are repaired postoperatively as shown in (D) and (E); whereas the dehiscence in layer (C) is not repaired as shown in (F).

**Fig 5:**
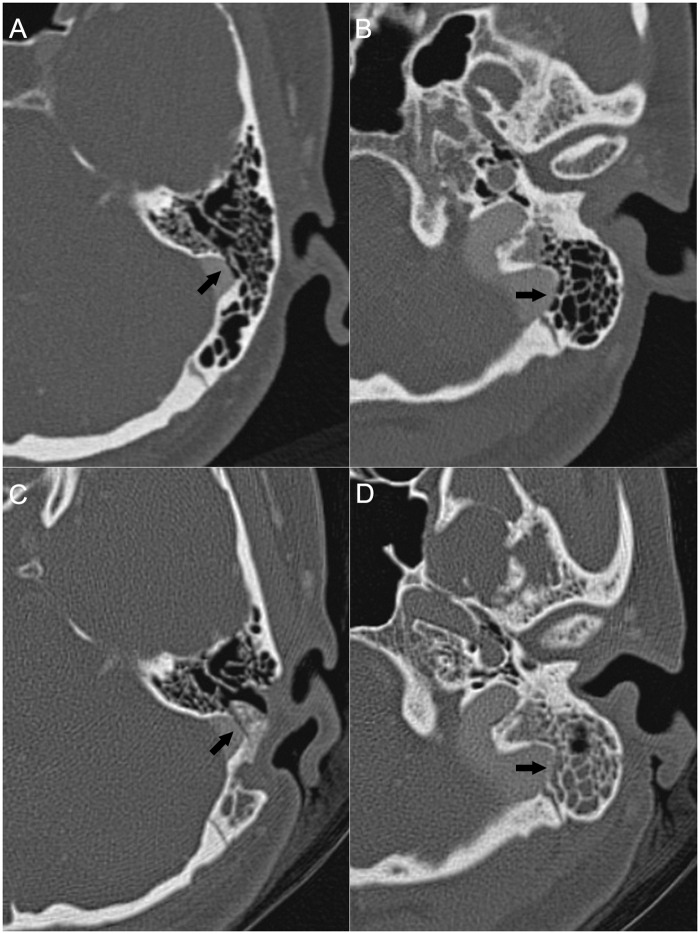
Preoperative (A, B) and postoperative (C, D) CTA images of a patient (Patient 16) with no change in PT following surgery. The layers in (A) and (B) correspond to those in (C) and (D), respectively. As shown in (C) and (D), the dehiscence in layer (A) is resurfaced, whereas that in the layer of (B) is not. A mastoid effusion is found postoperatively (D).

A total of 10 patients with partial resolution (n = 4) or no change (n = 6) underwent postoperative CTA. One or more dehiscences (range: 1–3) were found but only part of them were well resurfaced in these patients (Figs 4 and 5, S1 Table).

## Discussion

In this study, 19 of 27 patients obtained complete or partial resolution of PT following sigmoid sinus wall reconstruction, and their postoperative THI scores were significantly lower than those obtained preoperatively. These data suggest that sigmoid sinus wall reconstruction is an effective treatment for patients with PT caused by SSWD. Furthermore, no serious surgical complications were found; thus, our sigmoid sinus wall reconstruction technique is safe. Our findings are consistent with those of previous studies [7, 8, 13, 19–21].

We further investigated the reason for the unsuccessful treatment outcomes. Comparison of the pre- and postoperative CTA findings in the patients without complete PT resolution revealed that several of the multiple dehiscences were only partially repaired. We concluded that surgical failure was the primary reason for an unsuccessful outcome; thus, it is imperative that all regions of the dehiscence are sufficiently exposed and resurfaced during surgery. Eisenman and colleagues describe a standardized surgical technique for SSD and/or SSWD. They reconstruct the bone defect of the sigmoid sinus wall with three layers: soft-tissue graft (temporalis fascia or neural acellular dermal matrix), hydroxylapatite cement, and autologous bone pate, from the innermost to outmost, and they obtain a higher success rate of surgery than that of our study [7, 8, 20, 21]. It indicates that the reconstruction method used in this study may not be creating an optimal sound barrier, resulting in some patients with partial or no resolution following surgery, and reconstruction with firm materials is recommended [24, 30].

Alternatively, it is possible that the surgery failed to completely correct the blood flow turbulence in some SSWD patients, resulting in partial or no resolution of PT. Both the bone defect and blood flow turbulence contribute to the pathogenesis of PT with SSWD; however, the precise mechanism involved in generating PT is still unclear. SSWD is likely to occur during pneumatization of the mastoid in older adolescence or the early adult years. And PT may arise when blood flow turbulence occurs, which can be generated by the increased irregularity of the vessel diameter or ipsilateral dominance of the drainage system [14, 31, 32]. Mechanical compression of the sigmoid sinus is an efficient method to correct the blood flow turbulence and cure PT [21, 25]; however, it may cause increased intracranial pressure following surgery if extensive compression is performed [26]. Studying the hemodynamic changes in the venous sinus of patients with SSWD may reveal mechanisms associated with the development of PT, and could be beneficial with regard to refining surgical techniques.

There were 20 patients with DSS in our study (Table 1). In the surgery group, patients with DSS had a higher rate of PT complete resolution than did patients with a normal sigmoid sinus (Table 2), suggesting that patients with DSS may be more responsive to the surgery. DSS is different from SSD. SSD manifests as a well-circumscribed sac where the vascular elements of the sigmoid sinus focally protrude into the adjacent mastoid area, whereas DSS generally shows smooth bulging of the sigmoid sinus into the mastoid area without diverticulum formation [14, 17, 22–24, 26]. DSS is also defined as a prominent sigmoid sinus, distensible sigmoid sinus, sigmoid sinus enlargement, or sigmoid sinus ectasia [14, 21, 23–26]. Although DSS is frequently found in patients with sigmoid sinus anomalies, there is no consensus term or diagnostic criteria for this condition. Thus far, a definitive classification scheme of sigmoid sinus anomalies is lacking and needs to be established in the future.

There was a large female predominance in this population, which is also found in SSD and/or SSWD patients of other studies [5–9]. This demographic feature resembles that of patients with idiopathic intracranial hypertension (IIH). Furthermore, several IIH imaging features occur more frequently in PT patients with SSD and/or SSWD than in non-PT individuals [32], indicating that IIH may be an underlying cause in some PT patients with sigmoid sinus anomalies. Possible reasons for this could be that the elevated intracranial pressure gradually erodes the osseous wall of the sigmoid sinus, resulting in wall dehiscence and diverticulum, or that the elevated intracranial pressure causes blood flow turbulence and leads to PT. The precise mechanisms between sigmoid sinus anomalies and IIH still need further investigation.

No significant differences in age, BMI or preoperative THI scores were found among patients with different outcomes, suggesting that these factors are not related to the surgical outcome. Most of the patients in the non-surgery group had constant PT, which suggests that likelihood of spontaneous resolution was low.

Although the CTA images revealed SSWD in patients 2 and 17, no dehiscence was detected during surgery. This outcome was likely caused by the partial volume effect of CTA. Because extensive pneumatisation of the temporal bone was found in all cases, it is difficult to distinguish between the extremely thin wall and a small dehiscence in CTA images. Thus, it is advisable to exclude patients with a small dehiscence to improve the efficacy of surgery. Standardized diagnostic radiographic criteria for SSWD have not been established; therefore, further investigation of the association between the development of PT and dehiscence size is necessary. PT was aggravated in Patient 17 at follow up. The reasons for this are unclear because the patient refused to undergo a postoperative radiological examination.

Our study provided some insights for the treatment of SSWD. First, we recommend careful reading of the CTA and DSA images before surgery to identify the number of regions, size and location of the dehiscence. Second, it is essential to eliminate the air cells around the dehiscence during surgery to ensure that the resurfacing materials fit closely to the sigmoid sinus wall and are stable. Furthermore, it is essential that all regions of the dehiscence are sufficiently resurfaced during surgery. Third, unlike the attenuated wall of the SSD, which is fragile and bleeds easily, the exposed segment of the sigmoid sinus was generally normal in patients with SSWD. Since there was little breakage or bleeding during surgery, local anaesthesia may be advantageous because the surgeon is able to obtain valuable feedback from the patients[25].

The retrospective design is a limitation of our study. Selection bias may have occurred because the study was conducted in a single institution and the results may not be representative of the entire population. However, because SSWD is a recently recognised cause of PT, the underlying pathogenic mechanisms are not yet well understood. Our results may provide insights into the diagnosis and treatment of SSWD. In the future, it is essential to carry out a multicentre perspective study of SSWD to clarify the pathogenesis and the diagnostic criteria, and to standardise surgical techniques.

## Conclusion

This study summarized the clinical features and surgical outcomes of PT patients caused by SSWD at our institute. Approximately 70% of patients obtained complete or partial resolution of PT after transmastoid sigmoid sinus wall reconstruction, and no serious complications were found following surgery. Based on the comparative analysis of pre- and postoperative CTA findings, we recommend that all regions of the dehiscences should be sufficiently exposed and resurfaced during surgery.

## Supporting Information

S1 Table Changes in SSWD in patients with partial or no resolution. (DOC)
