# Supplementary material for: Sigmoid Sinus Wall Reconstruction for Pulsatile Tinnitus Caused by Sigmoid Sinus Wall Dehiscence: A Single-Center Experience
Source: PLoS One. 2016 Oct 13;11(10):e0164728. doi: 10.1371/journal.pone.0164728 (PMC5088000; doi:10.1371/journal.pone.0164728)
Supplement: S1 Table — (DOC) [file pone.0164728.s001.doc]

| **Surgery outcome** | **Patient No.** | **Before surgery** | | | |  | **After surgery** | | | |
| --- | --- | --- | --- | --- | --- | --- | --- | --- | --- | --- |
|  |  | **Number of SSWD** | **Area 1**  **(mm^2^)** | **Area 2**  **(mm^2^)** | **Area 3**  **(mm^2^)** |  | **Number of SSWD** | **Area 1**  **(mm^2^)** | **Area 2**  **(mm^2^)** | **Area 3**  **(mm^2^)** |
| Partial resolution | 9 | 1 | 43.59 | 0 | 0 |  | 1 | 1.92 | 0 | 0 |
|  | 22 | 1 | 23.03 | 0 | 0 |  | 1 | 2.185 | 0 | 0 |
|  | 4 | 2 | 3.09 | 6.43 | 0 |  | 1 | 2.93 | 0 | 0 |
|  | 11 | 2 | 28.63 | 20.985 | 0 |  | 2 | 3.715 | 8.44 | 0 |
| No change | 2 | 1 | 6.86 | 0 | 0 |  | 1 | 6.765 | 0 | 0 |
|  | 6 | 1 | 5.195 | 0 | 0 |  | 1 | 5.925 | 0 | 0 |
|  | 23 | 1 | 13.115 | 0 | 0 |  | 1 | 9.6 | 0 | 0 |
|  | 7 | 2 | 5.255 | 7 | 0 |  | 2 | 4.28 | 7.075 | 0 |
|  | 25 | 2 | 9.655 | 8.47 | 0 |  | 1 | 23.085 | 0 | 0 |
|  | 16 | 3 | 14.94 | 2.62 | 4.02 |  | 2 | 0 | 2.7 | 3.365 |

**S1 Table. Changes in SSWD in patients with partial or no resolution**
